# Partaking in the global movement for occupational mental health: what challenges and ways forward for sub-Sahara Africa?

**DOI:** 10.1186/1752-4458-6-15

**Published:** 2012-09-08

**Authors:** Olayinka Atilola

**Affiliations:** 1 Department of Psychiatry, University College Hospital, Ibadan, Nigeria

## Abstract

There is an ongoing global movement for the entrenchment of occupational mental health as an integral part of occupational health and safety schemes. Aside from being a fundamental human right issue, this move has been demonstrated to be of cost-benefit in terms of workplace productivity and general economic growth. Despite being among the regions most prone to the human and economic repercussions of work-related mental health problems by reason of her socio-economic circumstance; sub-Sahara Africa is yet to fully plug into this movement. With a view to make recommendations on the ways forward for sub-Sahara Africa, this paper examines the current state of and the barriers to effective occupational mental health policy and practice in the region.

## Introduction

There is an ongoing global discourse on the issue of mental health in the workplace and the focus had been on how countries are ensuring the prevention of mental stress in the workplace; facilitating unhindered access to curative and restorative care for work-related mental health problems, and incorporating workers who suffered mental health injuries in employee compensation schemes [1,2]. Though this discourse appeared to have started as a reactionary response to criticisms of the long standing relegation of mental health in the global debate on workplace health and safety; it has evolved over the years into a global movement for prioritization of occupational mental health. The impetus for this movement includes the advent of research evidence, that work-related mental health insults may be a bigger problem than physical injuries which had dominated the global occupational health and safety discourse. This was in view of sundry research findings that showed that mental health problems in the workplace affects productivity; encroach on profitability and take a higher toll on the economy than physical health problems.

Notable among such research findings is the observation, in a study which used data from close to 400,000 employees drawn from six major employers in the United State of America (USA), that productivity costs due to mental health problems in the year 1999 was almost double that of physical health problems [3]. Also, results from a survey in a major corporation in USA showed that healthcare and disability costs for mental health problems were at least as much as that for physical health problems [4]. It was also reported about that same time that mental health problems in the workplace encroach more on productivity, profitability and organizational stability than physical health problems [5]. This finding was attributed to higher utilization of health resources, higher risk of absenteeism, and higher risk of job-turnover among sufferers of work-related mental health problems compared with physical health problems [5]. It has also been found that absenteeism in the workplace is attributable to mental stress and anxiety than physical illnesses or injury [6], and that the burden of productivity loss due to mental health problems goes beyond the issue of being absent from work. For instance, there is research evidence that an overwhelming majority (81%) of the lost productive time to depression in particular was due to ‘presenteeism’ (reduced work performance) rather than absenteeism [7].

Other influential research findings that stimulated the current global discourse on prioritization of mental health in the workplace include the finding that, despite the often restrictive legal and administrative framework in most jurisdictions, the cost of compensation for mental health disability is similar or higher than that of physical disability claims among workers. Recent statistics showed that mental health disability claims constitute 35%, 40% and 56% of workplace disability claims for the year 2007 in Netherlands, United Kingdom and Austria respectively [8].

However, much of the dividends of the global discourse on mental health in the workplace had been largely more visible in the developed countries of America, Europe and Asia where it has served as a springboard for sweeping reforms in occupational mental health policies and practice. This was characterized by heavy investments in primary prevention of mental health problems in the workplace as a strategy to improve productivity and reduce future healthcare and disability costs. Literature and model of best-practices in primary prevention of occupational mental health is dominated by corporations in and authors from developed countries e.g. [9-11]. This is further evident of the differential uptake across regions, of the lessons learnt from research revelations on the global cost of un-prevented and un-treated mental health problems in the workplace. In a recently published meta-analysis of effectiveness of primary mental health promotion interventions in the workplace, all the 22 studies available for review were from America, Europe and Asia [12]. The same goes for secondary occupational mental health promotion, as most diagnostic tool and treatment guidelines for mental health problems in the workplace are developed in developed countries e.g. [13,14]. Similarly, the debate to fully liberalize and standardize the provisions for mental stress in national employees’ compensation frameworks as a tertiary occupational mental health strategy, is still largely a developed world affair e.g. [15-17].

Paradoxically, by reason of their socio-economic circumstance, the developing regions of the world are the most prone to the human and economic repercussions of work-related mental health problems. For instance, most countries in sub-Sahara have the awkward combination of high birth rate, low life expectancy and poor socio-economic development [18]. This scenario has left many sub-Sahara African countries with a predominantly youthful population and high rates of youth unemployment [19]. The combination of high youth population and poor infrastructural capacity for industrialisation in many countries of sub-Sahara Africa also create a situation for exploitative and unhealthy working conditions. In such settings, the prevalence of occupational mental health problems are expected to be very high, bearing in mind that the social disadvantage associated with poor human development itself can also constitute a perpetual mental health stressor in the first instance.

Furthermore, sub-Saharan Africa is among the worst-hit regions by the ripple effects of the global economic crisis [20]. It is expected that the risk of work related mental health problems in the region will increase. This is in view of likely sweeping change in workplaces’ policies and practice on employee welfare towards more stringent ones, in response to the economic impact of the global economic meltdown. This can then set a vicious cycle in which productivity continues to drop due to the damaging effect of the desperate cost-cutting measures by employers, on the mental well-being and productivity of workers. A large proportion of employees may also be compelled to voluntarily resign on mental health grounds with attendant wastage of skilled human resource.

With the current challenging economic and human development situation in sub-Sahara Africa, the region can not afford further erosion in her human-resource capacity for development. Therefore, one of the ways to maximise human capita for sustainable development in sub-Sahara Africa include national work-place policy thrusts toward promoting mental wellbeing in the workplace. It has been argued that advocacy and research on the issue of occupational mental health in developing countries is an urgent priority [21]. There is also a compelling need for developing countries, not to only join in the ongoing global debate by initiating national and regional discourse on the issue of mental health in the workplace, but to begin the necessary sensitization and reforms along this line. Beyond the ‘developing countries’ generalisation, regional advocacy is more feasible and wieldy in a well defined region. The region of sub-Sahara Africa comprise of countries with fairly homogenous socio-economic, political and cultural milieu which can dictate common barriers to uptake of contemporary innovations in occupational mental health.

Therefore, from the point of view and in the experience of an indigenous mental health practitioner, the rest of this paper examines the current state of and challenges to occupational mental health in sub-Sahara Africa. Practical ways of deepening occupational mental health policies and practice in the region will also be discussed. It is hoped that the discourse will provide an angle to the barriers to and ways forward for evolving occupational mental health policy and practice in sub-Sahara Africa, and stimulate further debate on the subject in the region.

### Occupational mental health in sub-Sahara Africa: current challenges and realities

With a view to examine the challenges to effective occupational mental health promotion and protection in sub-Sahara Africa and to speculate on the current state of preventive, curative and restorative occupational mental health in the region; this section of the paper examines the current challenges and realities in sub-Sahara Africa.

### Socio-economic and political circumstance of work in sub-Sahara Africa

Majority of workplaces in sub-Sahara Africa are faced daily with the challenges occasioned by the problematic infrastructural and social framework for industrial growth and productivity in the region. Notable among these are unstable power supply, inefficient transportation and port systems, non-transparent business environments, unfriendly tax-regimes, and political instability with its attendant policy inconsistencies. Among other social and infrastructural problems, the total energy generated by the entire sub-Sahara Africa (excluding South Africa) as at 2008 was a meager 28 GW which was about the capacity of Argentina [22,23]. The ports system in many sub-Saharan African countries are also not structured to promote business efficiency as they are poorly equipped, operate below installed capacities, and fall behind international standards in terms of regulatory and technological frameworks [24]. Furthermore, the business environment in sub-Sahara is also not transparent as majority of the countries in the region are on the lower half of the 2011 Corruption Perceptions Index [25]. Doing profitable business in this kind of setting will ordinarily require a lot of cost-control measures among which, unfortunately, is the reduction of workers welfare package to the barest minimum.

In addition, the revenue bases of many of the big corporations and major employers of labor in developing countries especially in sub-Sahara Africa often rival the gross domestic product (GDP) of the countries where they operate [26]. This peculiar situation often create a situation whereby big corporations have undue overbearing influence on the polity and the legislative/judicial machinery of economically and politically weak countries [27]. A plausible scenario is for the big corporations to manipulate such influences to further their economic interests through unbridled employees’ exploitation, taking undue advantage of the famously high level of youth unemployment and the deficient employee’s protection systems in the region [28].

Moreover, in a justified effort at boosting their share in a globalising economy, governments of most developing countries have had to embrace the concept of the so-called Export Processing Zone (EPZ). The EPZ is an industrial zone with special incentives put in place to attract foreign investors, in which imported materials undergo some degree of processing before being re-exported [29]. There are currently about a hundred EPZ in sub-Sahara Africa concentrated more in countries like Gabon, Ghana, Kenya, Lesotho, Mali, Mozambique, Nigeria, Zimbabwe, Madagascar, Mauritius and South Africa [30]. Part of the often ‘unwritten’ special incentives for the EPZ in many of these countries includes exemption from part of the labor code, including occupational health and safety (OHS) regulations [31,32]. As a result, many of these EPZs have become hot-beds for violation of workers rights. This is yet another source of challenge to occupational health, including mental health, in sub-Sahara Africa [32].

Beyond the infrastructural challenges for productivity and the socio-political influences on work and work situations, there are other work-indicators in sub-Sahara Africa that may increase the risk of exploitative work and hinder the entrenchment of decent-work. For instance, sub-Sahara Africa has the second highest Employment-to-Population Ratio (EPR) in the world [33,34] and account for up to 12% of the global workforce [35]. For a resource-poor region with the lowest productivity per person-employed in the world [36], the high EPR may not necessarily mean that the economy of sub-Sahara Africa is generating more work. Rather, it may suggest a scampering for any form of work among the populace in view of the poor state of social security coverage in the region [37,38]. In other words, the primary consideration of the average prospective employee in sub-Sahara Africa may not necessary be the degree of decency of such works but the fact that socio-economic realities may dictate that they continue to work. It has been mooted that the main labor issue confronting sub-Sahara Africa is not unemployment as it were, but the quality, productivity and decency of employment [35].

In addition, from Lesotho and Mozambique, through South Africa and Swaziland to Zambia and Zimbabwe; there are reports of large proportions of employees being engaged under exploitative working conditions in the name of casualisation of labor [34]. A recent report showed that in the last 20 years, the proportion of Kenyan employees employed as casual workers in the formal sector has risen by 25% [39]. There are also reports from Nigeria that up to 40%-90% of the workforce of some sectors of the economy is made up of casual workers [40,41]. Aside the menace of casualisation of labor in sub-Sahara Africa, there are other realities that may erode on the promotion of mental health of workers in the region. The high level of poverty and lack of social welfare safety net in many sub-Sahara African countries creates a situation of high dependency on working family member [42]. Yet, the remuneration of workers in the region is poor with almost 90% of workers earning less than US$2 per day [42]. This scenario is expected to place a lot of strain on the employee, in addition to the stress of unhealthy and exploitative working condition.

Another peculiar characteristics of the labor market in sub-Sahara Africa is the high prevalence of informal employment, which accounts for up to 70% of regional employment [35,43,44] and as high as 90% in some countries of the region [45]. Informal sector works are generally characterized by deprivation of commensurate remuneration, poor job security, inadequate or absent social protection and denial of power of collective bargaining [46-49]. Beyond the informal sector, other common form of ‘engagement’ in sub-Sahara Africa even if illegal includes a high prevalence of forced labor and child labor. Sub-Sahara Africa has the highest population per capita of child laborers among the regions of the world [50] and the third highest rate of forced labor [49].

### State of and barriers to occupational mental health promotion in sub-Sahara Africa

Though unavailability of relevant databank will make the generation of accurate data on the current state of workplaces’ strategy for mental health promotion in sub-Sahara Africa difficult; the realities on ground suggest that many employers of labor in the region would have taken the mental wellbeing of their employees as opportunity cost for cost-control. Though the last assertion can be said to be speculative, the speculation is not without a sound basis. For instance, when faced with the combination of a highly in-efficient and expensive business environment and poor employee productivity; physical and mental well-being of employees is a plausible opportunity-cost for profitability. The high prevalence of casualised work in sub-Sahara Africa reflects the tendency for employers in the region to want to shelve the perceived burden of welfare and well-being of employees. This is because the main motivation for casualisation of labor is the exemption from commitments to worker’s welfare and well-being.

The tendency for employers of labor in sub-Sahara Africa to want to trade the physical and mental wellbeing of employees for a larger balance-sheet is further complicated by the weak and resource-constrained institutions of both labor administration and law enforcement in the region [42]. The combination of lack of unemployment-benefits and a large population of the working-poor in sub-Sahara Africa [51] also ensures that a large proportion of workers are unable to liberate themselves from indecent or forced labor [42,52]. The fact that employers continues to have a regular supply of casual or other indecent forms of work in the region supports the notion that the high EPR in sub-Sahara Africa may not be driven by job creation, but by the precedence of basic survival over considerations of the decency of work. A situation whereby employees continue to work out of economic, social or exploitative compulsion, and where the labor market is flooded continuously with prospective workers; is ordinarily expected to reduce the motivation of employers to invest in the health, nay mental health of employees. This is more so, when the employers are also struggling to keep afloat, given the socio-economic realities on ground.

Closely linked with forced labor is the large informal economy in sub-Sahara Africa. This is because persons working in the informal sector as well as those in forced servitude or working as a child are all more likely to work under conditions where optimal promotion of occupational health (and occupational mental health specifically) can not be envisaged. They are also often beyond the reach of existing employees’ social protection schemes [53,54]; more likely to experience unfair and exploitative treatments at work [42] and at higher risk of being involved in a poor psycho-social working environment [55].

**Figure 1:**
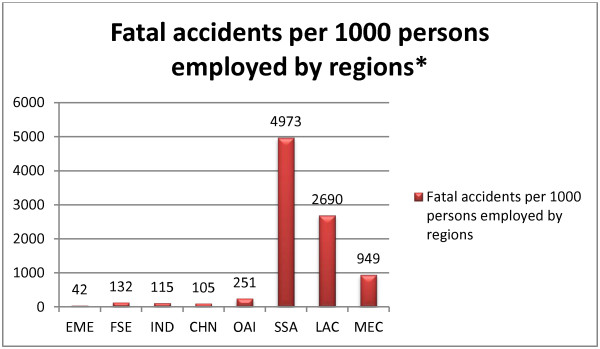
Fatal accidents per 1000 persons employed by regions*. EME = Established Market Economies, FSE = Formerly Socialist Economies, IND = India, CHN = China, OAI = Other Asia and Islands, SSA = Sub-Saharan Africa, LAC = Latin America and the Caribbean, and MEC = Middle Eastern Crescent. *Computed by the author based on the report by Hamalainen et al. [56]

Even for the few who work in the formal labor market, there are reports that occupational hygiene in sub-Sahara Africa is at the lowest end of global standards [43]. As shown in Figure 1, the region also has the highest incidence of violent and fatal accidents per 1000 employed in the world, based on the estimated figures by Hamalainen et al. [56]. Though these reports depict more of the safety of the physical work-environment, there is an interconnection between physical safety and mental health. The degree of real and perceived safety of the workplace has a direct bearing on the mental health of employees. For instance, witnessing or being in the vicinity of a violent injury or death, as well being a survivor of a violent trauma is associated with lifelong psychological distress and can provoke a wide array of mental disorders [57].

### State of and barriers to management of work-related mental health problems in sub-Sahara Africa

Despite the obviously high risk of mental stress in the workplace in many countries of sub-Sahara Africa, the current framework for management of work-related mental health problems is still poor in the region. Low level of mental health literacy, poor attitudes towards and low prioritisation of mental health issues among community dwellers in sub-Sahara Africa has been identified as among the barriers to mental health care systems in the region [58,59]. The effect of this has been seen in the delivery of occupational mental health care in some countries in the region. In a study of 200 work managers in Port Harcourt (Nigeria), while many of them believed in the need to use different methods of stress alleviation as a means of ensuring continuous wellbeing in the workplace; less than half of them endorsed mental health services like counseling and other psychological interventions as a veritable means of stress reduction [60]. Also, preliminary data from a survey conducted to examine the knowledge of, attitude towards and prioritization of workplace mental health issues among a cohort of HR managers drawn from different companies in Nigeria revealed poor indicators. For instance, majority would not want to employ a person with an established mental disorder, will not be willing to reinstate an employer who suffered mental disorder back to his/her previous positions after ‘recovery’ and did not prioritize mental health seminars among the health education strategies in the workplace.

While many countries in sub-Sahara Africa are yet to domesticate most of the International Labor Oganisation (ILO) conventions on occupational safety and health [28], the few that have done so may also lack prerequisite infrastructure for enforcement. Moreover, Gureje and Alem (2001) described ignorance of and lukewarm attitude towards mental health issues among policy makers in sub-Sahara Africa [61]. Therefore, it is possible that the few countries that have domesticated the ILO conventions on occupational safety and health, ignorance of and prejudices against mental health issues may have prevented the full incorporation of mental health into occupational health and safety schemes.

 Beyond ignorance and attitude, the professional capacity for occupational health, which is a cornerstone issue in ensuring a healthier workplace is also seriously lacking in sub-Sahara Africa. For instance as at 2003, human capacity for professional occupational health services was reported to be skeletal in most countries of the region, and non-existent in countries like Ghana, Zambia and Malawi [62]. In a region like sub-Sahara Africa where there is a severe shortage of mental health professionals to meet even the burden of basic mental health care [58,59], a near absence of professional capacity for occupational mental health and safety in the region can be safely speculated. The implication of this is that even when workplaces are willing to incorporate mental health and safety into their occupational health scheme, lack of trained human resource in the field may be a de-motivating factor for such intent.

### State of compensation for mental health injuries in sub-Sahara Africa

One of the major ways of alleviating the personal suffering of individuals who suffered psychological injuries in the course of employment is inclusion of compensation for such in the national framework for employee’s compensation. Unfortunately, while most countries of sub-Sahara Africa have some legislative provisions for physical injuries and disabilities sustained in the course of work, scarcely any of them have a distinct provision for mental stress sustained in the course of work. Although South Africa has a legal provision that allows for application for retirement from work on mental health grounds [63], the Compensation for Occupational Injuries and Diseases Act of South Africa (as amended in 1997); the Work Injury Benefits Act of Kenya, and the Injuries Compensation Act of Gambia just to mention a few, has no specific provision for compensation for mental health injury. One country that has taken a step in this direction, albeit very recently, is Nigeria with the inclusion of a distinct provision for compensation for mental stress in the newly promulgated Employee Compensation Act (ECA) of 2010 [64].

From the foregoing, it is obvious that a mixture of social, cultural, economic, political and infrastructural factors has hindered the evolution of occupational mental health as an aspect of holistic OHS strategy in sub-Sahara Africa. This affectation cuts across all aspects, including promotion, management and restorative aspects of occupational mental health.

### The ways forward for occupational mental health in sub-Sahara Africa

This section provides an angle to the direction that efforts at improving occupational mental health in sub-Sahara Africa should follow. The multi-faceted approach adopted in the current discourse assigns roles for employers and human resource (HR) managers, labor law policy makers, the body mental health professionals, and State governments in the region.

### Employers and human resource managers

With a very high EPR, it is obvious that there is a huge scampering for jobs among community dwellers in sub-Sahara Africa. The tendency then is for employers to jettison mental health promotion in the workplace on the ground that replacement of ‘burnt-out’ employee would not be tasking. This will however be without cognizance of the costs of hiring, training, and the inertia of employee adaptation. High prevalence of un-prevented and unmanaged mental stress in the workplace affects the mental health of the worker and impugns on productivity and organizational advancement through absenteeism, high job turnover and loss of productive time in conflict resolutions [65]. Common mental health problems in the workplace like depression and anxiety disorders are however preventable and are very amenable to treatment, with objective and subjective improvements in work performance after treatment [66,67]. Therefore, advocates of occupational mental health in this region need emphasize the treatability at least, of occupational mental health problems and that doing so is in the best interest of the establishment.

Employers of labor and human resource managers in sub-Sahara Africa must pay attention to the mental health of their workers, not only because doing so is in conformity with extant guidelines for OHS, but because it ensures continued productivity. One of the simplest ways to achieve this is to invest in the welfare of employees as an incentive to drive productivity rather than viewing employees’ welfare as an opportunity cost for sustaining profitability. Innovative approaches include the establishment of a mental-health and welfare sub-committee comprising of representatives from the worker’s body, human resource unit, and mental health safety experts, as part of workplace occupational health and safety policy [68]. Such committee will be responsible for the evaluation and monitoring of compliance with pre-set occupational mental health goals and recommend specific actions for implementation [68]. A good practice in sub-Sahara Africa which has almost become effectively defunct is the Psychosocial Services Unit (PSU) which used to be integrated into most big corporations in South-Africa in the 1990s, and provide mental health services to workers [69]. The then PSU comprised of occupational social workers, occupational therapists, psychiatrists, psychologists and staff of the HR department [69]. Such framework, which used to work, can be resuscitated or adapted in any sub-Sahara African context to suit the realities of today.

Other practicable measures to enhance workplace mental health in this region include stress reduction through flexibility and predictability of work-hours, adherence to terms of employment, and institutionalization of holidays, recreation, stress management training seminars and employee assistance programs. The workplace is gaining popularity as an appropriate setting for provision of small scale mental health services for employees [70,71]. Therefore, scheduled opportunities for on-site counseling, psychosocial support and treatment of mental health problems can be created in the workplace [68]. In developing regions like sub-Sahara Africa, it may be unrealistic to view workplace stress as being distinct from general environmental stress. In any case, even in developed countries, there is evidence that up to 25% of employees who attend on-site counselling programs do so for issues outside of work [72]. Therefore, a workplace help desk for counseling and support of employees in sub-Sahara Africa on sundry extraneous stress-inducing issues like family, social, financial and health problems will be ingenious.

### State governments

Though there is currently no reliable data on the contribution of occupational mental health to regional productivity in sub-Sahara Africa, the region currently has the least global productivity output per-person-employed in the world [33]. Eroding this further through employee exploitation and disregard for employees’ health is the last thing that the region can afford now. Governments should therefore enact and vigorously enforce legal instrument to outlaw casualisation of labor and any form of exploitative and dehumanising practice in the workplace. A regional example is the recommendation by the Ministry of Labor and Social Security of Zambia to amend the Employment Act of the country to outlaw casualisation of labor in response to public outcry (culled from The Post of Zambia, 31^st^ Aug 2010). In addition, an accessible, efficient and anonymous complaint and redress system should also be put in place for victims.

Beyond this, as a proactive step towards ensuring a better feasibility of adequate occupational mental health in the region, governments of sub-Sahara African countries need to urgently stem unemployment and underemployment as bulwark for employee exploitation in the first place. It has been argued that unless there is an improvement in the unemployment and under-employment statistics in sub-Saharan Africa in the next few years, the region may lose capacity for needed economic and human emancipation [73]. There are two ways to go in this regard: create employment and cushion the effect of unemployment. Provision of unemployment-benefits may not solve the underlying problem of poor job creation, but it will reduce the compulsion to accept indecent jobs. This may compel employers to improve on their welfare packages for employees.

 To create employment, the current natural-resource (non-agric) driven monolithic economies of most sub-Sahara Africa must be diversified. It has been recommended that natural-resource driven economies should also develop other sectors like agriculture and manufacturing whose growth are more tied to job creation [74]. Arguing in the context of sub-Sahara Africa where agriculture still accounts for up to 65% of employments, the ILO has argued that economic development in the region can use some structural changes, but that this was not possible without development of the agricultural sector [75]. The economic transformations (including job creation) that accompanied the revolution of the animal hide industry into the second largest export earner in Ethiopia [33] provides a good example of how agriculture can transform the fortunes of countries in sub-Sahara Africa.

In addition, to evolve into a modern economy which can keep her burgeoning youth population gainfully employed, governments of sub-Sahara African countries must tackle the issue of infrastructural backbone for productivity. There is an urgent need for State governments to improve the power situation in the region by bridging the current funding gaps in the power sector and to initiate the needed reforms in terms of the operating performance of power installations of the region [22]. Similar reforms are needed in the region’s ports to facilitate easy inflow and outflow of raw materials and other commodities. In addition, there is a compelling need to sincerely address the issue of corruption and wastages. Institutions of law making and law enforcement should be strengthened and countries must show more commitment to the independence of national anti-corruption agencies.

Poverty and social inequalities have been cited among the key drivers of forced labor and other indecent work in developing regions like sub-Sahara Africa [46,48,49]. Therefore, as bulwark to the vulnerability of workers to exploitation, governments need to come up with sustainableand realistic poverty reduction and wealth-redistribution strategies. Such strategies should include wealth redistribution through progressive tax systems, responsive social protection schemes and well established social services [42]. Setting a truly ‘minimum’ wage, understood as the least income that can sustain a decent living and aggressively monitoring its universal application in all sectors of the economy, should be part of governments’ priorities also.

### Labor policy makers

Policy makers in labor matters in sub-Sahara Africa needs to embrace the issue of mental health as a matter of human right that it is. The United Nations General Assembly Resolution 46/119 recognized that any exclusion of any kind on mental health grounds violates human rights [76]. Therefore all forms of non-incorporation of mental health into health systems, including occupational health systems, should be seen as exclusion. There is also a need to develop capacity in all aspects of occupational health, including mental health, in the region. This will help to raise the critical human resource needed to drive policy and practice development in the region. In other jurisdictions, research and capacity building in occupational health has been strengthened through the establishment of national institutes. An example is the National Institute for Occupational Safety and Health (NIOSH) in the USA, established as far back as 1970. The US NIOSH is responsible for research and capacity building in occupational health through the funding of and involvement in research, policy development and trainings in the field. There is currently no country in sub-Sahara Africa with the kind of framework of the NIOSH of USA. However, the recent foundation-laying of the National Occupational Safety and Health Institute (NOSHI) in Nigeria, which was patterned in principle after the NIOSH of USA, is a step in the right direction. According to Nigeria’s Minister of Labor and Productivity, the institute hopes to serve as the research and training hub for OHS in Nigeria and sub-Sahara Africa. The institute hopes to achieve these through conduct of regular risk assessment into work environments and processes in the country, and conduct regular evaluation of industrial hygiene among other functions. Other sub-Sahara Africa countries may want to take a cue from this laudable project.

But beyond that, the NOSHI of Nigeria should truly envision herself as the hub for occupational health and safety capacity in the region by incorporating regional awareness and collaboration efforts into her agenda from the outset. More importantly, it is pertinent for NOSHI of Nigeria, and other similar institutes which may later be established in the region, to note that incorporation of mental health services in the agenda from the outset is the surest way of ensuring non-exclusion. This may include the incorporation of a department of occupational mental health in the establishment and including a directorate of occupational mental health in the administrative structure from the outset [68]. The sum of these efforts will be the entrenchment of occupational mental health in the occupational health systems in the region.

Despite all its challenging implications for successful OHS policies, the informal sector also constitutes a major component of the gross national income (GNI) of most countries in sub-Sahara Africa. The sector accounts for as high as 60% of the GNI in countries like Nigeria, Tanzania and Zimbabwe [77]. The sector is also a major gender equalizer in the labor market in the region as a large proportion of women who could not penetrate the formal sector are engaged in the informal sector in sub-Sahara Africa [78]. In light of this, it has been argued that in addition to efforts at 'formalising’ the informal economy as panacea to OHS challenges in the sector, policies should also be geared towards eliminating the problems of informality of employment while preserving its role in advancing the economy and bridging gender inequalities in employment [35]. In this regard, governments and policy makers need to commit themselves to combating deliberate informalisation of employment like casualisation, baseless contractual employment and outsourcing, through sound legal frameworks and vigorous law enforcement [42]. In the same vein, labor policy makers in sub-Sahara Africa need to come up with innovative programs that can build the capacity of companies operating in the EPZ to comply with labor and OHS laws, as well as provide incentives for compliance [79].

In addition, as a matter of adherence to international global best practices and compliance with the tenets of equity and social justice; labor policy makers in all sub-Saharan African countries should begin a process to amend their respective workmen’s compensation schemes to include compensation for mental injuries. Though there is yet to be a global consensus on the scope of coverage for occupational mental stress compensations, sub-Sahara African countries should first take the bold step using the basic principle of providing equitable and inclusive compensations for both acute and chronic mental health problems which arose in the course of work. However beyond this, it is equally important to ensure compliance with such provisions as mental health statutes on their own can only give legal basis for equity, they don’t translate to ‘equity in the real sense’ without active enforcement [80].

Nigeria has a provision for mental stress in her newly promulgated ECA. This can serve as a local reference point in the quest of other sub-Sahara African countries for inclusion of compensation for mental health injury in their respective workmen compensation laws. However, policy makers in sub-Sahara Africa who may want to use the provisions for mental stress compensation in Nigeria’s ECA as template of their own should be cognizant of the fact that the provisions therein, just like any such provisions globally, has been found wanting in certain aspects. The import of this development is that in such quests, policy makers need review current trends in the rapidly evolving field. A comprehensive review of the strengths and shortcomings of the Nigeria’s ECA as well as guidelines for ensuring successful implementation among stakeholders has been done [81,82].

### Mental health and allied professionals

Being the custodian of mental health care in all regions, mental health professionals in sub-Sahara Africa also have a role to play in the quest for better occupational mental health in the region. Mental health professionals in this region should be at the fore front of advocacy for occupational mental health through workshops and seminars organised for and in collaboration with HR managers. Mental health campaigners in the region can also adopt the workplace as one of the setting for awareness creation, and liaison centre for mental health service provision. This will improve mental health literacy among employees and may divulge mental health from the widely reported myth and prejudices [61,83]. A division of occupational mental health can also be created within the African Association of Psychiatrists and Allied Professions to stimulate research and advocacy in this field among mental health professionals in the region.

In view of the limited number of mental health practitioners in sub-Sahara Africa [58], focusing on training of designated occupational mental health specialists in the region may not be the right step. Rather, it should be advocated that occupational mental health issues, including short industrial attachment, be incorporated into the training of Psychiatrists and allied professionals in the region. This will ensure that every mental health practitioner in sub-Sahara Africa have basic practical knowledge of occupational mental health and is able to provide services in this regard. For the same reason of human resource shortage, a role can also be carved for traditional mental health practitioners in occupational mental health in the community level. Such roles could include traditional trauma-focused care for persons who may have been witness to severely traumatic events in the workplace. A good adaptable example is the cleansing and purification ritual used in the traditional treatment of emotional problems among child soldiers in Mozambique [84]. These rituals derived from the cultural notions that exposure to violence constitutes a form of pollution and uses symbolism in an effective way to reduce emotional symptoms of traumatic exposure. The same principle can be adapted in the traditional management of the emotional consequences of exposure to violent injury and death in the workplace. The extractive industries in sub-Sahara Africa where accidents are common and where most junior employees are sourced from local communities readily comes to mind.

## Conclusion

The framework for occupational mental health in sub-Sahara Africa is such that it is still far from international standards but there is a slow but progressive effort towards improvement. Occupational mental health schemes still continues to evolve in many countries of the region and the continued quest for an holistic best-practice is in view of the importance of workers mental health on continued productivity. The need to maximize the human resource of sub-Sahara Africa as an elixir for economic emancipation continues to provide sound rationale for further investment in the physical and mental well-being of employees in the region. Achieving this will require coordinated efforts involving employers of labor, governments, labor law policy makers and the body of mental health and allied professionals in the region.

## Abbreviations

ECA: Employee compensation act; EPZ: Export processing zone; GDP: Gross domestic product; GNI: Gross national income; HDI: Human development index; HR: Human resource; ILO: International labor organisation; NIOSH: National institute for occupational safety and health; NOSHI: National occupational safety and health institute; OHS: Occupational health and safety; PSU: Psychosocial service unit.

## Competing interests

The authors declare that they have no competing interests.
